# Bioengineering *Caulobacter vibrioides* for Xylanase Applications in the Bakery Industry

**DOI:** 10.3390/microorganisms13102367

**Published:** 2025-10-15

**Authors:** Bruna Simioni, Paula Maria Carneiro Rocha, Adriano Fávero, José Luis da Conceição Silva, Rinaldo Ferreira Gandra, Alexandre Maller, Marina Kimiko Kadowaki, Rita de Cássia Garcia Simão

**Affiliations:** 1 Laboratório de Bioquímica Molecular, Centro de Ciências Médicas e Farmacêuticas, Universidade Estadual do Oeste do Paraná (UNIOESTE), Cascavel 85819-110, PR, Brazil; bruna_sm95@hotmail.com (B.S.); jose.silva3@unioeste.br (J.L.d.C.S.); 2 Laboratório de Enzimologia e Tecnologia das Fermentações, Centro de Ciências Médicas e Farmacêuticas, Universidade Estadual do Oeste do Paraná (UNIOESTE), Cascavel 85819-110, PR, Brazil; alexandre.maller@unioeste.br (A.M.); marina.kadowaki@unioeste.br (M.K.K.); 3 Laboratório de Micologia, Laboratório de Análises Clínicas, Ensino, Pesquisa e Extensão (LACEPE), Hospital Universitário do Oeste do Paraná (HUOP), Rua Tancredo Neves, 3224, Cascavel 85806-470, PR, Brazil; rinaldo.gandra@unioeste.br; 4 Laboratório de Bioquímica de Microrganismos, Centro de Ciências Médicas e Farmacêuticas, Universidade Estadual do Oeste do Paraná (UNIOESTE), Cascavel 85819-110, PR, Brazil

**Keywords:** enzyme technology, gene expression, fermentation, use of agro-industrial waste, aquatic bacterium, synthetic biology

## Abstract

The present study investigated the impact of genetic engineering strategies to produce a cell-free xylanase for applications in the baking industry. The *xynA1* gene from the nonpathogenic bacterium *Caulobacter vibrioides* was integrated into the pAS22 vector with a xylose-inducible promoter and introduced back into the bacteria, resulting in the creation of the BS-*xynA1*. This construct exhibited substantial secreted xylanase 1 (XynA1) activity, reaching 17.22 U/mL, and a specific activity of 278.64 U/mg after an 18 h growth period with 0.3% (*v*/*v*) xylose plus 0.2% (*w*/*v*) corn straw. RT-qPCR analysis confirmed that higher xylanase activity in *C. vibrioides* cells was correlated with increased transcription of the *xynA1* gene in the induction medium. Moreover, BS-*xynA1* cells coexpress other enzymes, including xylanase 2 (XynA2), cellulase, pectinase, α-amylase, β-glucosidase, β-xylosidase, and α-L-arabinosidase, at low levels (≤2 U/mL). In vitro comparison of cell-free xylanases from BS-*xynA1* with three commercially available xylanase-containing mixtures commonly utilized in baking protocols revealed its superior specific activity (163.4 U/mg) across a broad temperature range (30–100 °C), with optimal performance at 50 °C. In practical baking tests, the addition of cell-free XynA1 led to a reduction in dough kneading time and increase in bread height compared to those of the control. Notably, the incorporation of XynA1 resulted in enhanced alveolar structure formation within the bread crumb. Specifically, the following changes were observed in the mass parameters compared to those of the control: an increase in extensibility, elasticity, and deformation energy, and subsequent improvements in strength. Additionally, XynA1 addition led to a reduction in toughness and toughness/elasticity index, indicating a reduction in the mass stiffness of the enzyme-treated bread. To date, this is the first successful application of recombinant XynA1 from *C. vibrioides* in biotechnological processes related to baking, underscoring the potential and prospects in the food industry.

## 1. Introduction

Xylan is a representative part of the lignocellulosic mass in plants, and its degradation involves the synergistic action of different enzymes, with endo-β,4-D-xylanases (E.C.3.2.1.8) and β-D-xylosidases (E.C.3.2.1.37) being the most important [1]. Endo-β-1,4-D-xylanases cleave the xylan main chain, producing xylo-oligosaccharides that can be converted to xylose by β-D-xylosidases [2]. In many biotechnological and industrial applications, bacterial xylanases have shown better performance than fungal xylanases [3,4]. The significant differences found were low cellulase activity, low cellulase stability, low cellulase activity in the alkaline range, and high cellulase thermostability, among others [5,6]. Moreover, superxylanases are very difficult to produce when all these features are combined, implying that most of the available bacterial xylanases have only one or two of these properties, leading industrial processes to depend on expensive and hazardous chemical processes or strategies for designing or engineering superenzymes [5,7].

In baking, xylanases are desirable because they improve dough extensibility and bread volume. Cellulase, on the other hand, can degrade cellulose and structural components associated with the wheat cell wall matrix, compromising the gluten network. This results in loss of gas retention, weakened dough, and, consequently, smaller-volume, worse-textured breads. Therefore, xylanases with low or minimal cellulase activity act selectively on the target polysaccharides (arabinoxylans), optimizing dough rheology without causing undesirable side effects on the flour’s technological quality. In short, this allows for better technological performance, to name a few, volume, texture, and tenderness, without compromising the integrity of the protein network that supports the dough structure [8].

Xylanases have been widely used in the baking industry for decades. They are employed in baking together with α-amylase, maleate amylase, glucose oxidase, and proteases. Xylanases breakdown hemicellulose in wheat flour, helping redistribute water and make the dough softer and easier to knead. During the bread baking process, they generate greater flexibility and elasticity, allowing the dough to grow. The use of xylanases in baking assures an increase in bread volume, better water absorption, and greater resistance to fermentation [9]. In the manufacture of cookies and crackers, xylanase is recommended to make them creamy and lighter and to improve their texture, palatability, and uniformity [1].

Xylanases are widely used as processing aids in the grain milling industry to produce flour. In this industry, xylanases are often referred to as hemicellulases or pentosanases. Thus, the term hemicellulase refers to the ability of xylanase to hydrolyze insoluble nonstarch compounds found in flour, while the term pentosanase indicates that the substrate for the xylan enzyme is composed of pentose monomers [10]. Therefore, xylanases can improve the quality of the baking process and bread dough. In *Bacillus* sp., for instance, the *xynHB* gene encoding xylanase was cloned and inserted into an antibiotic-free vector and integrated into the yeast genome. The yeast strain A13 overexpressing xylanase was applied in baking and showed a reduction in the time required for kneading and an increase in bread dough height and diameter [10].

Several species, primarily from the *Bacillus* genus (*B. pumilus*, *B. circulans*, *B. amyloliquefaciens*, *B. stearothermophilus*, *B. halodurans*, *B. subtilis*), as well as *Streptomyces*, *Geobacillus*, *Actinomadura*, *Saccharopolyspora*, and *Thermotoga*, are described as producers of thermostable and hyperthermophilic xylanases [11]. Other genera, such as *Micrococcus*, *Staphylococcus*, *Paenibacillus*, *Cellulomonas*, *Arthrobacter*, and *Microbacterium*, also contribute to the diversity of these enzymes. In practical applications, the xylanase from *Aureobasidium pullulans* NRRL Y-2311-1 has shown efficacy in gluten-free cookie formulations, improving the rheological properties of the dough and the softness of the product [12].

Bacterial xylanases mostly belong to the GH10 and GH11 glycosyl hydrolase families, according to the CAZy (Carbohydrate-Active enZYmes Database) classification [13]. These enzymes present broad functional and structural diversity. GH10 xylanases are generally enzymes with higher molecular mass, more open active sites, and the ability to act on highly substituted xylans, while GH11 xylanases have a more compact structure, high specificity, and greater catalytic efficiency on linear xylans, which reflects different adaptive strategies and biotechnological applications [14,15,16]. Recent studies demonstrate that variants of these families exhibit additional properties, such as thermostability, halo-tolerance, and resistance to adverse industrial conditions, expanding the potential for use in sectors such as baking, biofuels and functional foods [14,15,16,17].

The genome of *Caulobacter vibrioides* Henrici and Johnson 1935 (*C. crescentus heterotipic*) [18] contains eight genes [19], which are directly involved in biomass degradation and are therefore relevant for biotechnological applications. Among these genes, only one encodes cellulase (*celA1*), two encode enzymes with endoxylanase activity (*xynA1-2*), and five encode enzymes with β-xylosidase activity (*xynB-xynB5*) [2]. This bacterium is an interesting source for studying hydrolytic enzymes since they are abundant and recurrent throughout the globe. According to the bacterial diversity metadatabase (BacDive) [20], there are currently records of 6141 *Caulobacter* isolates on the planet, of which 2002 isolates were identified in water bodies, 1503 in soil, 969 in plants and 1667 in different animals, of which 731 were identified in humans [20,21]. *C. vibrioides* has traditionally been considered nonpathogenic and assumed to be nontoxic to humans, making it a promising bioengineering vector for environmental remediation, the food industry, and medical applications [22].

The *C. vibrioides xynA1* gene [3] was previously characterized through the overexpression and purification from *E. coli*. Different properties were defined, highlighting that the recombinant enzyme XynA1 exhibited an optimal pH equal to 6 and peaked at 50 °C. XynA1 has demonstrated remarkable thermal stability for biotechnological applications, retaining 80% of its activity over a span of 4 h at 50 °C. Studies using thermostable xylanases with an optimum of 50 °C are justified by their greater stability and efficiency under typical baking thermal conditions, ensuring better technological performance and a lower risk of contamination in the process [23]. Then, the present study introduces a novel process approach in which a new strain was engineered in a way where the cloned *xynA1* gene expresses the XynA1 enzyme under the control of a xylose-inducible promoter and not by the native promoter. This approach allows, in the controlled conditions of a process, only the cloned *xynA1* gene to be more expressed by the new strain since its native promoter is not regulated by xylose [24,25].

As mentioned before, *C. vibrioides* possesses a second xylanase encoding by the *xynA2* gene as XynA1 is characterized as GH10 [4]. Although XynB2 displays 50% activity at temperatures ranging from 40 to 90 °C, it has an optimum pH of 8 and less than 35% activity at pH 6. In addition to these biochemical characteristics that do not suit applications in baking processes, the *xynA2* gene is regulated by xylose [24], which would not allow exclusive control of its expression by xylose addition, as is possible with the *xynA1* gene. Thus, in a context in which both genes, *xynA1* and *xynA2*, are expressed, it is clear whether the resulting enzymatic activity comes from the expression of the XynA1 or XynA2 enzyme, simply because they have different biochemical analysis parameters. So, in the present work, the expressed and secreted *C. vibrioides* XynA1 was incorporated into flour and applied throughout the baking processes.

## 2. Materials and Methods

### 2.1. Bacterial Strains and Growing Conditions

The strains and plasmids used in the present work are described in Table 1. *Escherichia coli* DH5α and S17 bacterial strains [26] were used for subcloning and conjugation, respectively. Both strains were grown at 37 °C and maintained at 4 °C in Luria–Bertani (LB) medium [27].

The *E. coli* strain containing the pAS22-*xynA1* [29,30] construct was grown at 37 °C and maintained at 4 °C in LB media supplemented with chloramphenicol (1 µg/mL). The bacterial strain *C. vibrioides* NA1000 was grown at 30 °C and maintained at 4 °C in PYE media (Peptone–Yeast Extract) containing 0.2% (*w*/*v*) bactopeptone, 0.1% (*w*/*v*) yeast extract, 1.7 mM MgSO_4_, and 0.5 mM CaCl_2_ (*v*/*v*). The BS-*xynA1* strain was isolated by conjugation with *E. coli*-pAS22-*xynA1* at 30 °C. BS-*xynA1* was maintained at 4 °C in PYE media supplemented with chloramphenicol (1 µg/mL) and nalidixic acid (20 µg/mL).

To induce the expression of the *xynA1* gene (XynA1) and other enzymes, the WT-pAS22 and *C. vibrioides* BS-*xynA1* strains were cultivated at 30 °C and 120 rpm in minimal medium (M2) (Na_2_HPO_4_ 12 mM, NH_4_Cl 9 mM, KH_2_PO_4_ 8 mM, MgSO_4_ 1 mM, CaCl_2_ 0.5 mM, FeSO_4_ 10 mM) [19] supplemented with 0.2% (*v*/*v*) glucose or 0.3% (*v*/*v*) xylose or 0.3% (*v*/*v*) xylose containing different agro-industrial residues at 0.2% (*w*/*v*) like corn straw (CS), corn cob (CC), hemicellulose from corn straw (HPM), wheat straw (WS), polisher residue (PR), vacuum cleaner residue from industry (WF), rice flour (RF), sugarcane bagasse (SB), rice straw (RS), and soybean residue (SR).

### 2.2. Cloning of the xynA1 Gene in the pAS22 Expression Vector

The DNA fragment corresponding to the *xynA1* gene (CCNA_02894) was isolated from the pJET1.2-*xynA1* construct [3] as described below. The *xynA1* gene of interest containing 1158 base pairs present in the pJET1.2-*xynA1* construct, along with the priming methionine and its stop codon, was digested with the restriction enzyme *Xho*I (Thermo Fisher Scientific^®^, Waltham, MA, USA). This enzyme cleaves this construct only to promote the linearization of the same construct. Then, a reaction with the enzyme DNA Blunting (Fermentas^®^, Burlington, NJ, USA) was carried out to obtain a noncohesive end, which allowed the subcloning process in the pAS22 vector at another noncohesive site in subsequent steps. With the gene still partially attached to the pJET1.2 vector at one end but containing a noncohesive end, it was digested with the restriction enzyme *Eco*RI (Thermo Fisher Scientific^®^). After digestion, the genetic material was resolved by 1% (*w*/*v*) agarose gel electrophoresis in TAE buffer. The 1793 bp gel band, corresponding to the *xynA1* gene and an untranslated portion of the pJET1.2 blunt plasmid that remained attached after the stop codon of the *xynA1* gene, was cut from the agarose gel using a scalpel and recovered with a DNA extraction kit (Pure Link Gel Extraction Kit—Invitrogen^®^, Carlsbad, CA, USA).

The plasmid used for *xynA1* expression [3] in *C. vibrioides* was pAS22 [29,30]. This plasmid has a xylose-inducible promoter, which allows for an increase in the expression of the cloned xylanase gene downstream of the promoter. Construction planning was based on experimental evidence that the *C. vibrioides xynA1* gene is not regulated by xylose according to transcriptomic analysis [24,25]. Furthermore, the gene was also cloned without the control of its original promoter. The increase in gene expression in this case can easily be accompanied by an increase in xylanase activity, dispensing with evidence for the accumulation of essential protein mass in the case of proteins that do not present measurable enzymatic function. Double plasmid digestion was performed with *Eco*RI and *Eco*RV restriction enzymes (Thermo Fisher Scientific^®^). This linearized vector was ligated to the DNA fragment corresponding to the *xynA1* gene (*Eco*RI/Blunt) with the aid of T4 DNA Ligase (BioLabs^®^, Cambridge, MA, USA). Subsequently, the pAS22-*xynA1* construct was used for transformation of the *E. coli* strain DH5α. Transformants were selected by growth in media supplemented with chloramphenicol (1 μg/mL), a resistance marker of the pAS22 vector. After the transformants were selected, the plasmid material was extracted by mini-plasmid preparation, double-digested with the restriction enzymes *Eco*RI/*Kpn*I, and resolved by 1% (*w*/*v*) TAE agarose gel electrophoresis to confirm the gene size.

### 2.3. Construction of the BS-xynA1 Strain

The pAS22-*xynA1* construct was used to transform the *E. coli* strain S17, which has conjugative functions. Transformants were selected by growth in media supplemented with chloramphenicol (1 μg/mL), the resistance marker of the pAS22 vector. After selection of the transformant, plasmid material was extracted by mini-plasmid preparation, double-digested with the restriction enzymes *Eco*RI/*Kpn*I and resolved by 1% (*w*/*v*) TAE agarose gel electrophoresis to confirm the recombinant size. *E. coli* S17 transformant strains containing the pAS22-*xynA1* construct were used for the transformation of the *C. vibrioides* NA1000 strain by conjugation. An aliquot of a fresh *E. coli* strain containing the pAS22-*xynA1* vector was mixed with twice the amount of fresh *C. vibrioides* cells of the NA1000 strain in solid PYE media. After 48 h of growth at 30 °C, samples of the bacterial cultures grown on the plate were streaked on a new solid PYE plate containing chloramphenicol (1 μg/mL), the pAS22 vector selection mark, and nalidixic acid (20 μg/mL), a selection mark of the NA1000 strain and not the *E. coli* strain, at 30 °C for 48 h. Isolated colonies grown on these plates were again streaked onto a new PYE plate containing the same amount of chloramphenicol and nalidixic acid. Microscopy analysis was carried out to confirm that only *C. vibrioides* cells were present in the medium. Aliquots of the BS-*xynA1* cells were then named BS-*xynA1* and stored at −20 °C and 80 °C for subsequent characterization. In parallel to the construction of the BS-*xynA1*, a control strain of the NA1000 strain was constructed via conjugation of the empty plasmid pAS22 to generate the strain control WT-pAS22.

### 2.4. Growth, Xylose Consumption, and Xylanase Production

*C. vibrioides strains* Cc-pAS22 and BS-*xynA1* were inoculated in 10 mL of liquid PYE medium supplemented with chloramphenicol (1 µg/mL) and nalidixic acid (20 µg/mL) and grown for 12 h at 30 °C and 120 rpm. When the cells reached the stationary phase of growth, they were diluted (OD_λ 600nm_ = 0.1) in a flask of 250 or 500 mL M2 medium supplemented with 0.3% (*w*/*v*) xylose and grown at 30 °C and shaking of 120 rpm. Every two hours of growth, a 2 mL aliquot of the growing culture was collected. From this volume, 1 mL was used to measure spectrophotometer cell growth, 1 mL was centrifuged at 15,000× *g* for 5 min at 4 °C, and the supernatant was reserved for extracellular xylanase activity analysis (optimizing for xylanase 1, pH 6 and temperature of 50 °C) in addition to the dosage of xylose consumption by the Orcinol method for pentoses [31]. Cell precipitates from centrifugation were frozen for further quantification of intracellular xylanase. The frozen cell pellet was lysed with 350 μL of 50 mM phosphate buffer, pH 6.0, under vigorous vortexing until thawing. The samples were kept on ice, and the overall activity levels of xylanolytic enzymes were determined. The assay and dosages were performed in biological and experimental triplicates, respectively.

### 2.5. Enzymes Production in Different Agro-Industrial Residues

Preinoculation and inoculation were performed as described in the previous section, but the M2 medium was supplemented with 0.3% (*v*/*v*) xylose and 0.2% (*w*/*v*) of different agro-industrial residues: corn straw (CS), corn cob (CC), hemicellulose from corn straw (HCS), wheat straw (WS), polisher residue (PR), vacuum cleaner residue from the industry (WCR), rice flour (RF), sugarcane bagasse (SB), rice straw (RS), and soybean residue (SR). All residues were prepared as described previously [25]. Cultures were incubated at 30 °C with shaking at 120 rpm for 18 h. Then, the samples were centrifuged at 15,000× *g* for 5 min at 4 °C. The supernatants were used to quantify XynA1 activity. The supernatant with the highest XynA1 activity was also characterized by the presence of xylanase 2, cellulase, pectinase, α-L-arabinofuranosidase, β-glucosidase, β-xylosidase, and α-amylase. Since the bacterium has two genes for xylanases (*xynA1*: CCNA_02894 and *xyn2*: CCNA_03137), two enzymes can be expressed under induced conditions. In these experiments, the NA1000 strain containing only the pAS22 (WT-pAS22) vector was used as a control.

### 2.6. Dosages of Different Enzymes

Reactions to verify XynA1 and XynA2 activity were performed using 1% (*w*/*v*) xylan from beechwood (Sigma^®^, St. Louis, MO, USA) as the substrate (50 mM sodium phosphate buffer, pH 6.0, for XynA1 and buffer McIvaine, pH 8.0, for XynA2) followed by incubation at 50 and 60 °C, respectively. Reactions to verify cellulase and α-amylase activity were performed using 1% (*w*/*v*) carboxymethylcellulose (CMC) as the substrate in 50 mM sodium citrate buffer (pH 5.5) and 1% starch as the substrate (*w*/*v*) in 50 mM sodium citrate buffer (pH 5.0), respectively, followed by incubation at 40 °C. Reducing sugars were measured using 3,5-dinitrosalicylic acid (DNS) [32]. Xylanolytic and cellulolytic activities were defined in U/mL as the amount of enzyme capable of releasing 1 μmol of xylose per mL of solution per min of reaction (U). The enzymatic activities of β-glucosidase, β-xylosidase, and α-L-arabinosidase were determined using ρ-nitrophenyl-β-D-glucopyranoside (ρNPG) and ρ-nitrophenyl-β-D-xylopyranoside (ρNPX) reagents and ρ-nitrophenyl-α-L-arabinofuranoside (ρNPA) (Sigma^®^), respectively, according to the adapted methodology described by Justo et al. [33], estimating the amount of ρ-nitrophenol (ρNP) released from the respective reagents. The total protein concentration was estimated by the Bradford method [34], which uses bovine serum albumin (BSA; Bio-Rad^®^, Hercules, CA, USA) as a standard.

### 2.7. Commercially Available Xylanases Versus Xylanase of BS-xynA1

Xylanolytic activity was also measured in different commercial xylanase mixtures available for application in the bakery industry. Among the variants tested were Spring CA 400 (produced by Granotec from Brazil S.A. Nutrition and Biotechnology, Araucária, PR, Brazil), Mega Cell 899, and Xylamax 292 (produced by Prozyn Industrial and Commercial Ltda, São Paulo, SP, Brazil). Enzymatic assays were performed as described in the previous section, with varying incubation temperatures (40, 50, 60, 70, 80, 90, and 100 °C) and pH 6. The same procedure was performed for the extracellular crude extract containing the *C. vibrioides* enzyme XynA1.

### 2.8. Test for Confirmation of the Absence of Viable Bacteria in Enzyme Extracts

The total enzyme extracts produced were centrifuged at 5000× *g* at 4 °C for 10 min. The centrifuged supernatant containing *C. vibrioides* XynA1 was sterilized with a 0.22 µm polystyrene vacuum filtration system containing sterile, pyrogen-free low-protein-binding polyether sulfone (Ciencor^®^, São Paulo, SP, Brazil). The complete absence of viable bacteria in the extracellular extracts was tested by plating a 100 µL aliquot of extracellular enzymatic crude extract into each of the three PYE media plates at 30 °C and LB at 37 °C followed by incubation for 48 h for microbial growth investigation. Aliquots of the filtrates and cell-free XynA1 were used for enzymatic assay testing after centrifugation and filtration to confirm the maintenance of xylanase activity.

### 2.9. mRNA xynA1 Gene Expression Analysis: RNA Extraction and RT-qPCR Assay

Analysis of *xynA1* and *xynA2* mRNA expression was performed by RT-qPCR using RNA extracted with Trizol (Thermo Fisher Scientific^®^) as indicated by the fabricant. The culture growth overnight in PYE medium at 30 °C and shaking at 120 rpm was diluted in M2 minimal culture medium containing different carbon sources separately: 0.2% (*v*/*v*) glucose (non-induced control); 0.3% (*v*/*v*) xylose; 0.3% (*v*/*v*) xylose + 0.2% (*w*/*v*) corncob (CC); and 0.3% (*v*/*v*) xylose + 0.2% corn straw (CS). WT-pAS22 and BS-*xynA1* strains were grown to OD_λ 600nm_ = 0.8–1.0 and total RNA was extracted. The primers *xynA1*-Forw (5′tgcgcgtttttcttggagcg3′) and *xynA1*-Rev (5′tcccgcgccggcgcggccttcag3′) were used to amplify cDNA from the *xynA1* gene, and the primers *xynA2*-Forw (5′atgtcacggctcacccgc3′) and *xynA2*-Rev (5′ctaccccgacagcacctc3′) were obtained to amplify the *xynA2* gene, both after global transcription with Reverse Transcriptase and random bacterial primers. cDNA was synthesized using 5 μg of RNA, 500 ng of random primers, and Superscript II, according to the supplier’s instructions (Thermo Fisher Scientific^®^). Approximately 200 ng of cDNA was used for quantitative amplification in the presence of 10 ρmoles of each oligonucleotide and 10 μL of Platinum SYBR Green (Applied Biosystems^®^, Waltham, MA, USA). The reference gene 16S rRNA constitutively expressed in *C. vibrioides* was used to normalize the quantities of target genes (16S-rRNA-Forw: gtacggaagaggtatgtggaac; 16S-rRNA-Rev: tatctaatcctgtttgctcccc), and the parental strain grown in glucose 0.2% was used as a calibrator in all tests, emphasizing that this sample was used as a basis for the comparative study of the expression results (ΔΔCT) [35]. All primers were designed using Primer Express Software version 3.0 (Applied Biosystems^®^). The presence of nonspecific products and the formation of dimers between the oligonucleotides were verified by the dissociation curve using the StepOne/StepOne Plus system software version 2.3 (Applied Biosystems^®^). The relative gene expression, ΔΔCT, was calculated by the ratio of the CT (cycle threshold) of the sample in the presence of glucose or test conditions (xylose and xylose containing agroindustrial residue). The data presented in this work are representative of two independent biological experiments performed in duplicate. The relative gene expression level was calculated from a group of replicates and expressed graphically. The variances of the data obtained were analyzed by the Anova test with a significance level of 99% (*p* < 0.01).

### 2.10. Software and IA Tools

Proteins and gene sequences were obtained from Kyoto Encyclopedia of Genes and Genomes (KEGG) [36]. Restriction map sequencing for cloning was carried out using Restriction-Map algorithms from the Bioinformatics Sequence Manipulation Suite [37]. Primer design was carried out using Primer Express Software version 3.0 (Applied Biosystems^®^).

Glycoside hydrolase families were found according to Carbohydrate-Active enZYmes Database (CaZY) classification [13]. XynA1 structure prediction was performed by alpha-fold generative AI version 3.0 [38].

The literature search was performed using the ScisSpace Research Platform (available at: https://scispace.com/copilot), which employs artificial intelligence to identify relevant articles through the personal signature of the corresponding author’s. The detection of similarities and originality in the present academic text was carried out using Turnitin software 2025 (https://www.turnitin.com) through an institutional signature. To survey bacterial biodiversity in the environmental context, the BacDive platform (Bacterial Diversity Metadatabase) was used [20]. 

The AlveoPC alveograph operates with Chopin’s proprietary software referred to as AlveoPC/Alveograph that is already installed in miniPC of the system to form the Chopin package (Chopin Technologies^®^, Willeneuve-la-Garenne, France) for acquisition, analysis the parameters and automatically emition of the test certificate. RT-qPCR data was analyzed using StepOne/StepOnePlus Software v2.3 (Thermo^®^, Waltham, MA, USA). Anova and Tukey’s test were performed using the software Microsoft Excel-v16.100.4/2025 (Microsoft Corporation^®^, Redmond, DC, USA). Some figures of this work (https://BioRender.com/g41tqq9 and https://BioRender.com/eee4z09) were created using the BioRender tool (https://www.biorender.com/). The remaining figures were created using Excel and PowerPoint through an institutional license for the Microsoft Office suite (Microsoft Corporation^®^).

### 2.11. Application of C. vibrioides Cell-Free XynA1 in Bread Formulation

To ensure food safety, the *C. vibrioides* BS-*xynA1* was grown in the absence of chloramphenicol since the cells maintained the pAS22 plasmid and xylanase production under the tested conditions for up to three consecutive days. The extracellular enzymatic extracts enriched with recombinant homologous XynA1 from *C. vibrioides* were filtered with a PES (polyestersulfone) vacuum filtration system of 0.1 μm and 500 mL capacity (431475-Corning^®^, Corning, NY, USA), and cell-free XynA1 was used in the baking assays. Therefore, it is certain that the final product used in the tests meets food safety standards. The xylanolytic activity of enzymatic extracts used as processing aids in the production of white wheat flour bread was investigated. This study aimed to evaluate the effects of these enzyme extracts on bread quality using a standardized loaf formulation adhering to the guidelines of Art. 661 of the Argentine Food Code.

The baking trials were meticulously executed in triplicate, employing carefully defined formulations. The core ingredients consisted of white wheat flour (as specified), sugar, salt, fresh yeast (Fleischmann from Brazil^®^, São Paulo, SP, Brazil), vegetable shortening (Coamo^®^, Campo Mourão, Brazil), and water. To explore the impact of enzyme extracts, two additional formulations were developed based on an experimental design in which the enzyme extract concentrations were manipulated. Adjustments in water content were made according to the volume of enzyme solution employed, ensuring consistency with the standard formulation. Refer to Table 2 for a breakdown of the ingredient quantities in each test formulation. The bread dough was mixed in a 25 kg ALI 25 1G3 bivolt Braesi semi-fast tilting mixer.

The dough preparation procedure included distinct phases, each of which contributed to the final bread characteristics: 1. Mixing (Phase I and Phase II): Initial ingredients, including flour, sugar, salt, and water (also incorporating enzymatic solution for tests T2 and T3), were combined in a trough maintained at a temperature of 4 to 6 °C. This mixture was blended at a constant speed (100 rpm) for 3 min. In Phase II, the blend welcomed vegetable fat and fresh yeast, and mixing was continued at 190 rpm until the desired dough consistency was achieved. 2. Division: The resulting dough was divided into uniform 500 g portions with precision, aided by spatulas and scales. 3. Modeling: The modeling phase involved a single pass through a specialized machine designed to roll, fold, stretch, and therefore homogenize the dough. This three-step process ensured an even dough texture and structure, promoting symmetrical bread formation during fermentation and baking. 4. Fermentation and Growth: The molded dough was shaped into standard forms and positioned within a controlled fermentation chamber set at 28 °C. This allowed for an exact fermentation period of 4 h, promoting optimal dough development. 5. Baking (Final Step): The fully formed, fermented, and grown dough was placed into a preheated oven set at 180 °C. Baking was carried out for 30 min. After baking, the loaves were allowed to cool to room temperature (25 °C). Bread height measurements were acquired using a measuring tape, and cross-sectional observations were made by halving the loaves to assess alveolar structure. Additionally, photographic documentation facilitated a comprehensive comparison between control loaves and loaves subjected to varying enzyme extract concentrations.

### 2.12. Alveograph Test: Evaluating Dough Viscoelastic Characteristics

The enzymatic impact on the viscoelastic properties of dough is assessed through the alveograph, a technique that emulates dough behavior during fermentation by replicating the formation of air pockets (alveoli) generated by yeast-released carbon dioxide (CO_2_). This assessment involves the analysis of various parameters to gauge the viscoelastic traits of different dough samples. Employing the Chopin Alveograph model, AlveoPC, crafted by Chopin Technologies (Villeneuve-la-Garenne, France) and adhering to the International Association for Cereal Science and Technology guidelines “URL: https://icc.or.at/icc-standards/standards-overview” (accessed on 19 August 2025), this test method provides precision to the characterization process.

For the enzyme addition experiment (Table 3), the enzyme extract volume was determined according to the ratio of enzyme extract per kilogram of flour as utilized in baking test T3. The control run, devoid of enzyme supplementation, served as a baseline. Consistency was maintained in both the control and test groups according to the following parameters in line with the AACC methodology recommendations: humidity, 14.4%; hydration, 50%; and B concentration, 15% H_2_O. The preparation involved diluting sodium chloride in sterile water to afford a 2.5% (*w*/*v*) saline solution, which was subsequently combined with the enzyme solution in the enzyme addition test. A total of 250 g of flour was introduced into the mixer at 24 ± 2 °C. The process begins, and within 20 s, saline solution is added. The mixing continued for one minute, encompassing the 20 s prior to saline addition. Following this, the mixer halts, allowing for the clearing of its sides using a spatula—a one-minute procedure. The mixture was mixed for an additional 7 min, resulting in a total of 8 min.

The mixer is then paused to alter the rotation direction of the paddle, leading to the division of the resulting dough into five uniform portions. These portions are placed within an alveograph rest chamber maintained at 25 ± 0.2 °C. Twenty-eight minutes after the initiation of dough mixing, the first portion is placed at the center of the stationary alveograph plate and coated with Vaseline. The lid is positioned and secured, and the plate undergoes two full turns within 20 s. A period of 5 s later, the lid and ring are removed, and the dough is allowed to inflate until a bubble formed. This sequence is repeated for the other four dough portions.

Parameters derived from the alveograph include toughness (P), which represents the peak pressure exerted during mass expansion (measured in millimeters); extensibility (L), which quantifies the length of the curve (measured in millimeters); and mass deformation energy (W), which signifies the mechanical work needed to expand the bubble until rupture, expressed as 10^−4^ J. The ratio of toughness to extensibility (P/L) offers insight into mass equilibrium, where P signifies the mass’s resistance to deformation, and L indicates mass extensibility.

### 2.13. Statistical Analysis

For the enzymatic treatment test within the bread formulation that precedes the alveograph analysis, the acquired data were subjected to analysis of variance (Anova) with a significance level of 99% (*p* < 0.01). Subsequently, a Tukey post hoc test was conducted to determine significant differences, maintaining a significance level of 99%.

## 3. Results and Discussion

### 3.1. Subcloning of the xynA1 Gene and Construction of the BS-xynA1 Strain

**Figure 1:**
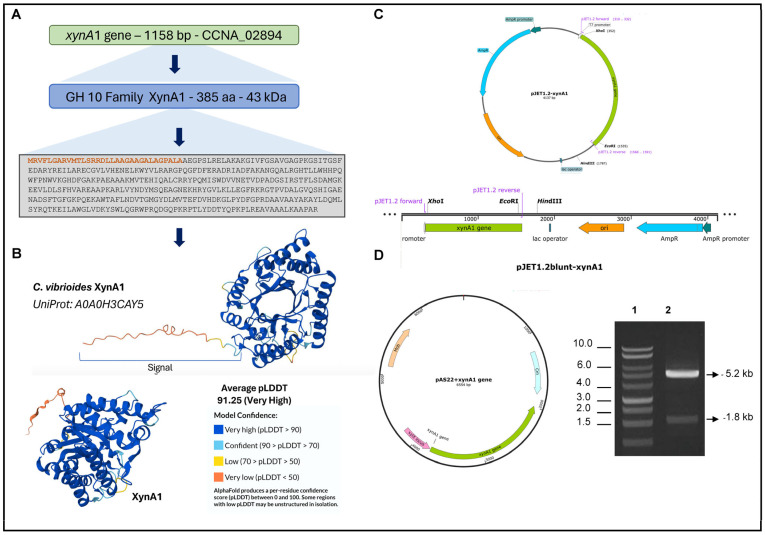
Strategies for engineering *C. vibrioides* strains that overexpress XynA1 in the strain BS-*xynA1*. (**A**) The *xynA1* gene (accession number: CCNA_02894) coding a xylanase that belongs to the GH10 family. The amino acid sequence predicted from the *xynA1* gene, and the amino terminal signal sequence highlighted in orange. (**B**) XynA1 structure prediction using Alpha-Fold Software version 3.0 [38]. (**C**) Genetic context of the *xynA1* construct in the pJet1.2-Blunt cloning vector. The pJet1.2-Blunt construct containing the *xynA1* gene was digested with *Xho*I and treated to produce blunt-end DNA. (**D**) Next, pJet1.2-Blunt-*xynA1* was digested with *Eco*RI, and the resulting fragment, containing the complete *xynA1* gene, was subcloned and inserted into the pAS22 vector (*Eco*RI/*Eco*RV) under the control of a promoter induced by xylose, generating the synthetic strain *BS-xynA1*, which expresses XynA1 when 0.3% (*w*/*v*) xylose is added. DNA agarose gel electrophoresis on 1% (*w*/*v*) TAE gel (1: molecular weight marker, 1 kb DNA ladder—Thermo^®^; 2: construction of pAS22-*xynA1* digested with *Eco*RI/*Kpn*I enzymes).

The *xynA1* gene coding a xylanase that belongs to the GH10 family according to CaZY is displayed in Figure 1A. The amino acid sequence predicted from the *xynA1* gene shows an amino terminal signal sequence highlighted in orange. In the present work, the amino acid sequence of XynA1 was submitted to structure prediction using Alpha-Fold [38]. The structure obtained displays a very high confidence score of pLDDT = 91.25 in most part of the structure protein except for the amino terminal end of the XynA1 that presents a signal sequence of secretion from the cytoplasm to the extracellular media (Figure 1B).

To construct the BS-*xynA1* strain, we generated a *C. vibrioides* strain harboring a single functional copy of the *xynA1* gene. This gene was placed under the control of an inducible xylose promoter [29]. The construction process involved assembling a DNA fragment of 1793 base pairs corresponding to the *xynA1* gene (Figure 1C). This DNA fragment was then combined with a portion of the pJET1.2Blunt vector situated after the *Eco*RI-digested stop codon (Figure 1C). The resulting fragment was inserted into the pAS22 expression vector, which had been predigested with *Eco*RI and *Eco*RV enzymes. Validation of the construct, named pAS22-*xynA1* (Figure 1D), was carried out by transforming it into *E. coli* DH5α. Successful cloning was confirmed through mini-plasmid preparation, followed by digestion with *Eco*RI and *Kpn*I restriction enzymes (Figure 1D). Subsequent resolution on a 1% (*w*/*v*) agarose gel in TAE revealed the presence of a distinct 5230 base pair band indicative of the plasmid pAS22. Another band at 1793 base pairs, corresponding to the *xynA1* insert, further confirmed the successful cloning of the expression vector.

The pAS22-*xynA1* construct was transformed into the *E. coli* strain S17 via a transformation process. The *E. coli* S17 strain harboring the pAS22-*xynA1* vector (Figure 1D) facilitated conjugation with the *C. vibrioides* NA1000 strain. Transconjugants of *E. coli*-(S17)-pAS22-*xynA1*/NA1000 were selectively cultured in PYE media supplemented with chloramphenicol (1 μg/mL) and nalidixic acid (20 μg/mL) at a temperature of 30 °C. The resulting clone was tested to determine the stability of the plasmid pAS22 within the *C. vibrioides* NA1000 strain after exposure to chloramphenicol, which was subsequently preserved at −80 °C. The derived strain, designated BS-*xynA1*, exhibited expression of the *xynA1* gene and xylanase activity. This strain was subjected to various enzymatic and bakery-related assays, as described in the following sections. All experiments were carried out using the NA1000 strain containing pAS22 empty plasmid as the control (WT-pAS22).

### 3.2. Growth, Xylose Consumption, and Xylanase Production of BS-XynA1 Strain

**Figure 2:**
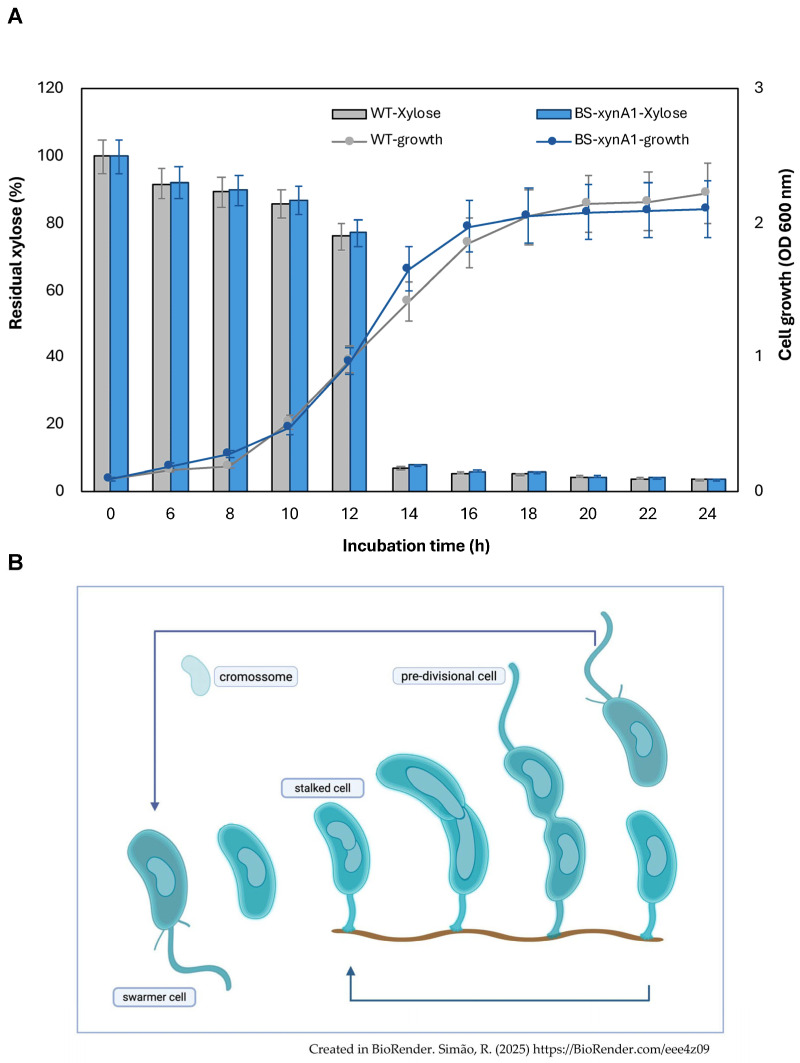
(**A**) Xylose consumption and cell growth curves of the wild-type strain (WT-pAS22) and the recombinant strain BS-*xynA1* in M2 media supplemented with 0.3% (*v*/*v*) xylose at 30 °C and 120 rpm. Cell growth (gray and blue continuous line) is expressed as O.D._λ=600nm_. The residual xylose content (gray and blue bars) is expressed as a percentage of the total initial sugar content. (**B**) Asymmetric life cycle of the bacterium *C. vibrioides*. (**B**) was created in BioRender. Simão, R. (2025) https://BioRender.com/eee4z09. For more details, see the text.

BS-*xynA1* was initially characterized for growth in minimal M2 medium supplemented with 0.3% (*w*/*v*) xylose (Figure 2A). The analysis of bacterial growth versus xylose consumption showed that they were very similar in both the parental control strain containing the empty plasmid (WT-pAS22) and the strain BS-*xynA1*. Xylose consumption occurred in both strains at the same rate as they advanced in different stages of the cell cycle (Figure 2A). In addition, the error bars of the growth curves of the WT-pAS22 and BS-*xynA1* strains overlap throughout the cultivation, indicating that both strains show similar growth rates (Figure 2A).

The bacterium *C. vibrioides* exhibits an atypical asymmetric cell division in a life cycle that lasts an average of 3 h in a defined culture medium [39]. In *C. vibrioides*, asymmetric division is a regular process in the life cycle, generating daughter cells with distinct morphologies and sizes. The life cycle begins with a motile cell that loses its flagellum, deconstructs its fimbriae, and begins the process of DNA replication by elongating. At the pole where the flagellum and fimbriae once stood, an adhesive stalk is assembled and, at the opposite pole, a new flagellum, thus forming a pre-divisional cell that will again give rise to two functionally and morphologically distinct daughter cells. The stalk and swarmer cell can have different average growth rates under conditions of abundant nutrients, despite sharing an identical genome and environment. In a standard culture such as the one presented in this work, there is a mixture of all cell types simultaneously (Figure 2B).

Both the parental (WT-pAS22) and BS-*xynA1* bacteria grow slowly in M2 minimal medium. Figure 1 shows a prolonged log phase of growth. This likely occurs for two distinct reasons. The first is that the growth medium is less rich, impacting the speed at which both strains reach the log phase of growth. The second reason may be related to the bacteria having to replicate the pAS22 plasmid, exhibiting typical slower growth in this condition.

**Figure 3:**
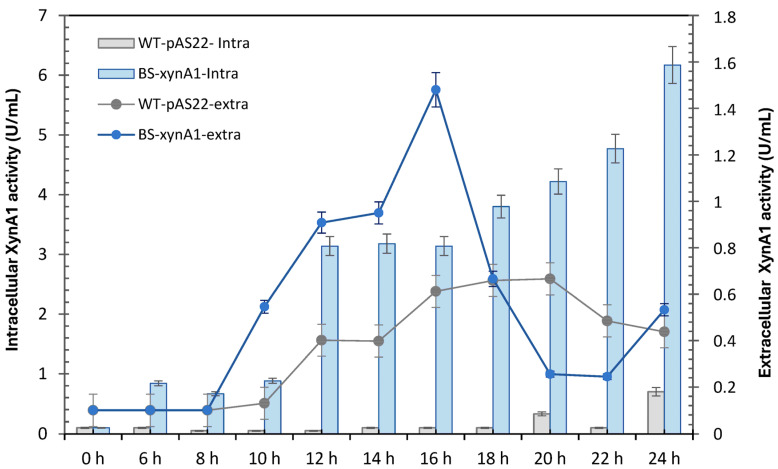
Intracellular xylanase activity of the wild-type strain (gray continuous line) and BS-*xynA1* (blue continuous line). Bacterial cells were grown in M2 media at 30 °C and 120 rpm. Extracellular xylanase activity of the WT (gray bars) and BS-*xynA1* (blue bars) strains of *C. vibrioides.* Bacterial cells were grown in M2 media supplemented with 0.2% (*w*/*v*) glucose (WT) and 0.3% (*w*/*v*) xylose (BS-*xynA1*) at 30 °C and 120 rpm for 24 h. Aliquots of the culture were removed, and xylanolytic activities were measured.

The control strains WT-pAS22 and BS-*xynA1* were grown in minimal media supplemented with 0.2% (*v*/*v*) glucose and 0.3% (*v*/*v*) xylose, respectively, for 24 h. The intracellular and extracellular xylanase activities of the strains were measured and are shown in Figure 3. Intracellular xylanase activity was greater in BS-*xynA1* than in the parental strain. Thus, the optimal yield was 6.16 U/mL, which is more than 4 times greater (1.485 U/mL) than that found in the extracellular media using the BS-*xynA1* strain after 18 h of xylose-induced cultivation (Figure 3). In the control strain, xylanase levels remained invariable between 16 and 20 h of growth and were lower than those in the BS-*xynA1* strain (Figure 3).

Even though both strains (WT-pAS22 and BS-*xynA1*) have the same growth profile, it is important to emphasize that the increase in enzymatic activity does not necessarily need to be related to the accumulation of cell mass; it can be derived from an increase or decrease in gene expression and even post-translational changes that can lead to enzymatic activation, among other factors. In this sense, it is possible to clearly see that even with similar growth profiles (Figure 1), the WT-pAS22 and BS-*xynA1* strains presented very different enzymatic activity profiles for XynA1 (Figure 2).

In addition, the data indicate that the BS-*xynA1* strain, which expresses homologous XynA1 in *C. vibrioides*, secretes part of the produced enzyme (Figure 3), since high extracellular enzymatic activity was verified.

**Figure 4:**
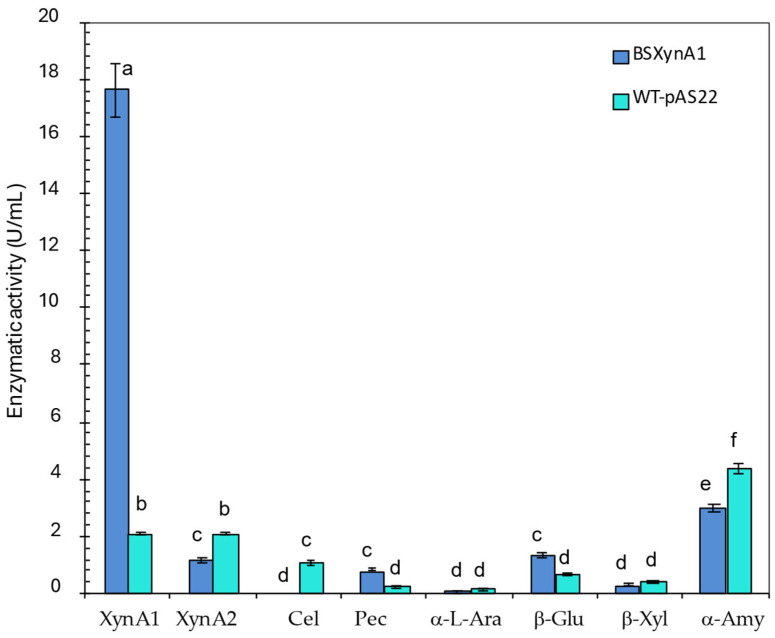
Enzyme activities produced by different *C. vibrioides* strains (WT-pAS22 and BS-*xynA1*) after growth in M2 medium supplemented with 0.3% (*v*/*v*) xylose and 0.2% (*w*/*v*) corn straw at 30 °C and 120 rpm for 18 h. Xylanase 1 (XynA1); Xylanase 2 (XynA2); Cel (Cellulase); Pec (Pectinase); α-L-Ara (α-L-Arabinofuranosidase); β-Glu (β-Glucosidase); β-Xyl (β-Xylosidase); and α-Amy (α-Amylase). Anova: degrees of freedom: 40; F calculated: 11.70; F critical: 3.12; *p* value: 5.45 × 10^−8^. The letters (a–f) show the statistical differences between groups considering a significance of 99% (*p* < 0.01). All results with the same letter are in the same group and therefore do not show significant changes. On the other hand, different letters indicate results that are statistically different.

Once again, it is important to emphasize that both xylanases present in *C. vibrioides*, XynA1 [3] and XynA2 [4], present different biochemical characteristics, to name a few, optimal pH and temperature, so this property is very reliable for differentiating the activity in the measurements prior to xylanases in bacteria. Considering the used biochemical parameters of pH and temperature to measure xylanase activity, only XynA1 from *C. vibrioides* could be detected at pH 6. However, XynA2 supports high temperatures like XynA1, and it presents an optimum pH of 8 and can display just 35% of its activity when incubated at pH 6 [4]. Even if XynA2 is expressed in the BS-*xynA1* strain, it can be safely stated that the predominant activity seen in Figure 4 refers to XynA1 and not to XynA2. Although extracellular xylanase levels are lower than enzyme levels within the cell, it is noted that part of the enzyme is secreted and allows its application in an easier and safer manner because it is cell-free and does not require expensive enzymatic purification steps.

Although XynA1 activity levels are predominant in the BS-*xynA1* strain due to increased expression from the P*xyl*-promoter, some XynA2 activity is invariably present, as the *xynA2* gene is not suppressed and, unlike *xynA1*, is upregulated by xylose. However, for the purposes of this study, the expression of both enzymes is favorable, even though XynA1 activity is more than 17-fold greater than XynA2 activity in the BS-*xynA1* strain (Figure 4).

### 3.3. Enzyme Quantification and RT-qPCR Assay Using BS-xynA1 Strain

To better characterize the extracellular extract produced by BS-*xynA1* and the parental strain (WT-pAS22), different enzymes were measured such as XyA1, XynA2, cellulase, pectinase, α-amylase, β-glucosidase, β-xylosidase, and α-L-arabinosidase (Figure 4). In the presence of 0.3% xylose (*v*/*v*) plus 0.2% (*w*/*v*) corn straw, the XynA1 activity was 17.22 U/mL, approximately 8 times greater than that present in the control strain (2.08 U/mL). On the other hand, the XynA2 activity was 1.16 U/mL (Figure 4), which is approximately 15 times lower than that observed for XynA1 in the BS-*xynA1* strain.

In a general way, all enzymes were very weakly coproduced with XynA1 in both strains. The activity of all enzymes was repressed in the BS-*xynA1* strain, except the proper XynA1 and β-glucosidase. These data suggest that XynA1 induction from the xylose promoter combined with the addition of a low concentration of corn straw as the carbon source leads to a peculiar activation of enzymes in general. The advantage of this strategy is the opportunity of isolation and control of the process because there is a higher activity of one enzyme in the extracellular extract, while other enzymes that are commonly coexpressed with xylanases are in low concentration (Figure 4). In consequence, it is possible to evaluate specific actions concerning enzymes with preponderant activity. This approach allows us to experimentally show the specific biotechnological role of XynA1 expression in bacterial enzyme cell-free extracts for the bread industry.

Under certain specific experimental conditions, the potential to achieve xylanolytic activities is higher than those observed in our current study. However, this heightened activity is often coupled with a diminished degree of control over other contributing factors, particularly other enzymes that are significant within the context of the fermentation processes of the bread industry. An illustrative example of this can be found in the work of Corrêa et al. [40]. In their research, they advocated for the optimization of β-xylosidase expression in *C. vibrioides* (strain NA1000) through the application of response surface methodology, utilizing corn cob as a carbon source. Within their optimized extract, a notably distinct profile of coexpressed enzymes emerges. Interestingly, the optimized conditions to produce bacterial β-xylosidases using a 3.5% corn cob substrate (393.36 U/mL) are associated with increased xylanase activity, which surpasses the levels observed in our present study (28.28 U/mL). This parallel increase in xylanase activity mirrors the pattern observed for β-xylosidases [40].

**Figure 5:**
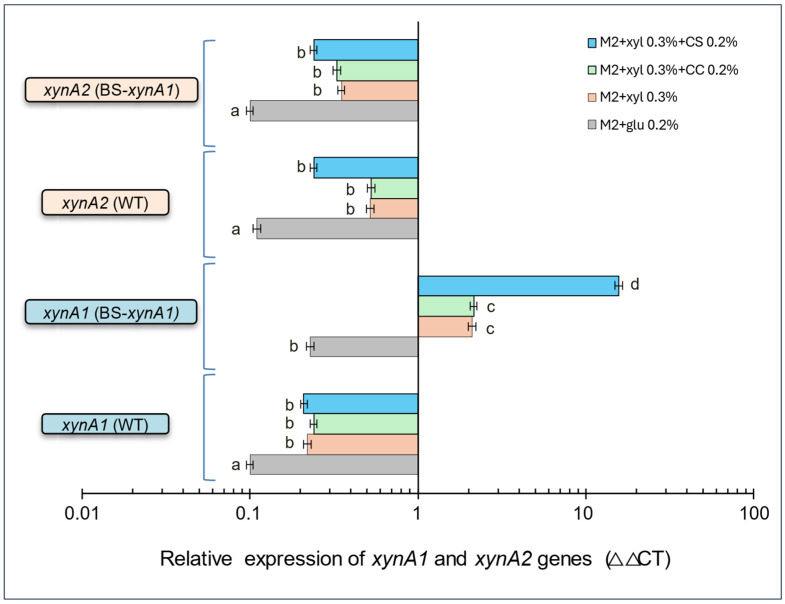
Relative gene expression of *xynA1* and *xynA2* genes in the *C. vibrioides* BS-xynA1 strain determined by quantitative real-time PCR (RT-qPCR). The *x*-axis shows data on a semi-log scale. The study aimed to understand how nutrient availability and growth conditions affect the expression of the *xynA1* and *xynA2* genes in *C. vibrioides* strains. RNA extraction and RT-qPCR protocols followed established procedures. Data normalization was performed using the 16S rRNA gene as an endogenous control, ensuring accuracy and reliability across treatments. Two independent biological experiments with two replicates each were conducted to ensure robustness and consistency. Relative gene expression levels were calculated for treatments under different media conditions. M2 minimum media containing glucose 0.2% (*v*/*v*), M2 minimum media containing xylose 0.3% (*v*/*v*), M2 minimum media containing xylose 0.3% (*v*/*v*) + corn cob (CC) (0.2% *w*/*v*), M2 minimum media containing xylose 0.3% (*v*/*v*) + corn straw (CS) 0.2% (*w*/*v*). Anova: degrees of freedom: 30; F calculated: 11,458.62; F critical: 3.70; *p* value: 2.98 × 10^−48^. The letters (a–d) show the statistical differences between groups considering a significance of 99% (*p* < 0.01). All results with the same letter are in the same group and therefore do not show significant changes. On the other hand, different letters indicate results that are statistically different.

Thus, to understand the enzymatic activity profile in the presence of corn straw, as shown in Figure 4, particularly regarding the discrepancy between the enzymatic activity measured for XynA1 and XynA2 enzymes, assays were conducted with total RNA from the BS-*xynA1* strain grown in the presence of 0.3% (*v*/*v*) xylose, either separately or in combination with corn cob (CC) and corn straw (CC). The total RNA samples of WT-pAS22 and BS-*xynA1* were used in the RT-qPCR assays with primers specific for the two genes *xynA1* and *xynA2*. The profile of enzymatic activities shown for XynA1 and XynA2 in the presence of CS (Figure 4) presents a parallel with the variation in the expression of the mRNA *xynA1* and mRNA *xynA2* (Figure 5).

Data also indicate that the variation in XynA1 occurs probably in response to P-Xyl induced by xylose in the construct, and by components present in the corn straw that positively affect the expression of the *xynA1* gene from its native promoter in the chromosome. So, the enzymatic activity is a response to the variation in the *xynA1* and *xynA2* gene expression in the presence of 0.2% (*w*/*v*) corn straw associated with 0.3% (*v*/*v*) xylose. Previous data for five genes encoding beta-xylosidases of the xylanolytic complex were also not affected by the addition of corn cob to the culture medium [40]. However, no tests were performed in the presence of corn straw, which appears to be effectively involved in increasing xylanase expression in the BS-*xynA1* strain. Additional experiments should be conducted in this regard to determine the reasons for this variation.

There was no significant variation in *xynA1* and *xynA2* gene expression in the presence of xylose or xylose associated with corn cob in the parental strain (WT-pAS22). However, in the BS*xynA* strain, *xynA1* was clearly induced in the presence of xylose (0.3%—*v*/*v*) plus corn cob (0.2%—*w*/*v*) and mainly in the presence of xylose (0.3%—*v*/*v*) plus corn straw (0.2%—*w*/*v*). The *xynA1* gene expression levels were significantly higher (*p* < 0.01), reaching more than 15 times the amount of messenger present in bacterial cells grown only in glucose, and it was higher than the levels of *xynA2* mRNA obtained in all other carbon sources compared (Figure 5).

### 3.4. XynA1 Production in Different Agro-Industrial Residues

**Figure 6:**
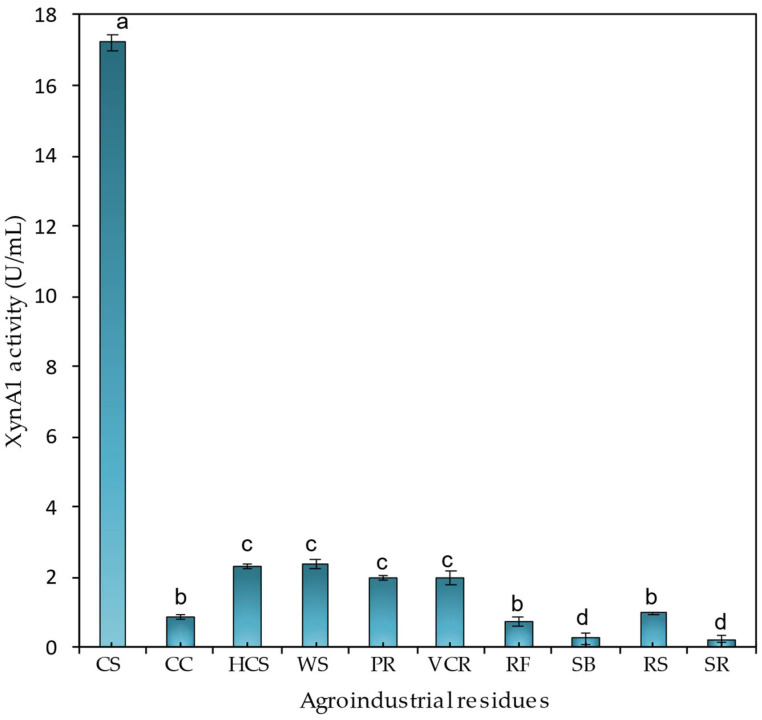
XynA1 production in the presence of different agroindustrial residues. *C. vibrioides* BS-*xynA1* strain cells were grown in minimal M2 medium supplemented with 0.3% (*v*/*v*) xylose and supplemented with 0.2% (*w*/*v*) agroindustrial residues: corn straw (CS), corn cob (CC), hemicellulose from corn straw (HCS), wheat straw (WS), polisher residue (PR), vacuum cleaner residue from industry (VCR), rice flour (RF), sugarcane bagasse (SB), rice straw (RS), and soybean residue (SR). The inoculum was generated by diluting the cells at the stationary phase to an O.D. λ of 0.1 at 600 nm in the same culture medium containing different carbon sources. Bacterial growth occurred at 30 °C for 18 h with agitation at 120 rpm. Anova data obtained: degrees of freedom: 20; F calculated: 941.43; F critical: 3.36; *p* value: 2.85 × 10^−24^ (*p* < 0.01). The letters (a–d) show the statistical differences between groups considering a significance of 99% (*p* < 0.01). All results with the same letter are in the same group and therefore do not show significant changes. On the other hand, different letters indicate results that are statistically different.

From the intriguing result shown in the previous section, the extracellular xylanase activity values obtained for each of the agro-industrial residues were tested to evaluate the most effective residue for xylanase production (Figure 6).

Among the residues tested, the highest xylanase activity (17.22 ± 0.22 U/mL) was obtained in medium supplemented with 0.2% (*w*/*v*) corn straw. The levels were significantly (*p* < 0.01) lower in the other residues, so in the presence of wheat straw and hemicellulose from wheat straw, there was practically no variation in xylanase activity (2.3 U/mL) or xylanase activity between the polished residue and the vacuum cleaner residue from industry (1.9 U/mL).

Different studies have reported that xylanase is an inducible enzyme secreted in media containing pure xylan or xylan-rich residues. Induction is initiated primarily by xylan. Some reports have shown the induction of xylanase by lignocelluloses such as wheat bran, rice straw, corncob, and sugarcane bagasse [5]. Xylose is the preferred carbon source for xylanase production by *Arthrobacter* sp. [41], and in *Streptomyces rameus*, dextrose is the best inducer of xylanase production [42,43]. Another study showed that xylanase induction appears to be species-specific in different bacterial strains [41].

### 3.5. Commercial Xylanases Versus Xylanase from BS-xynA1

**Figure 7:**
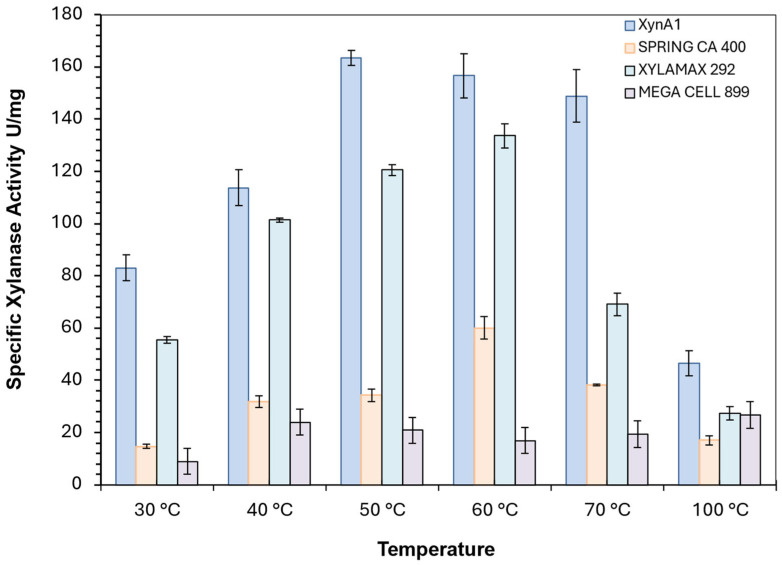
Comparison between the specific activity (U/mg) of different commercial xylanases, spring CA400 (yellow bars), Xylamax 292 (green bars), Mega Cell 899 (rose bars), and cell-free XynA1 from BS-*xynA1* strain (blue bars). Anova data obtained: degrees of freedom: 184; F calculated: 14.35; F critical: 2.74; *p* value: 7.88 × 10^−15^ (*p* < 0.01).

Commercial enzymes commonly used in baking flours in industry are spring CA400, Xylamax 292, and MegaCell 899. All of them were compared with cell-free XynA1 in enzymatic dosing assays. As a comparative parameter, the values of the specific activities (U/mg) of commercial enzymes and the XynA1 enzyme present in the cell-free extract obtained after BS-*xynA1* strain culture filtration were adopted. The highest specific activity observed for XynA1, 163.397 U/mg, was at 50 °C, which coincides with the optimal XynA1 enzyme temperature previously reported [3] (Figure 7).

In fact, XynA1 overexpression resulted in the highest specific activity (above 148 U/mg) at temperatures ranging from 50 to 70 °C in comparative studies. In addition, the specific enzymatic activity remained high at levels that are suitable for diverse applications in the manufacturing industry at all temperatures tested (30–100 °C) (Figure 7). These data show a certain robustness for *C. vibrioides* XynA1 in the large temperature range in which it has activity, demonstrating the qualities necessary for a baking enzyme (Figure 7).

### 3.6. The Absence of Bacteria in the Enzyme Extracts Was Tested

The experiment involved testing enzyme extracts for the presence of bacteria using two different growth media: PYE at 30 °C and LB at 37 °C. After an incubation period of 48 h, no bacterial growth was observed in either of the media, as indicated by the lack of visible bacterial colonies on the agar plates (Figure S1). This suggests that the enzyme extracts were free from viable bacteria. This outcome could be interpreted as a successful result, indicating that the XynA1 extracellular is a cell-free enzyme.

### 3.7. Application of XynA1 in Bakery

In the present assay, three groups were considered: “kneading,” which corresponds to the time taken to beat the dough until it reaches the veil point; “fermentation,” defined by the height of the bread after fermentation; and “baking,” determined by the height of the bread after baking. Each group was evaluated with the three previously described treatments, each analyzed in triplicate, totaling nine replicates per group. The data obtained (Table 4) indicated significant differences between the samples within each group, with a 99% significance level, a result confirmed by analysis of variance (Table 4).

The calculated F value greater than the critical F value confirms the existence of inequality between the measurements obtained. Additionally, Tukey’s post hoc test was applied. The least significant difference (LSD) defined by this test also demonstrated statistically significant differences between the analyzed groups. In other words, whenever the difference between the means of two modules (kneading/fermentation; fermentation/baking and kneading/baking) exceeded the LSD value, a significant difference was observed between the groups.

Table 4 clearly demonstrates the existence of significant differences related to dough kneading time. Samples subjected to an additional 1 min of kneading showed statistically significant differences, while those kneaded for 1 min less (T2 and T3) showed no significant variation. In the kneading/cooking modular relationship, a significant difference was also observed depending on the kneading time (3.1; 1.4).

In general, the Tukey test showed that both kneading time and enzyme addition influence the results of the kneading/fermentation and kneading/baking modular (2.2) relationships. Longer kneading times imply greater energy consumption, highlighting the importance of optimizing this parameter to reduce energy expenditure in baking (Table 4). Factors such as flour characteristics, water volume, and the addition of carbohydrate-degrading or -debranching enzymes also contribute to reduced kneading time [44].

From another perspective, the significant difference also depended on the enzyme concentration added. In treatment T2, in which a lower amount of enzyme was used compared to treatment T3, a significant difference was observed between the modules tested (1.4) in both enzyme treatments. An inverse effect, still dependent on enzyme concentration, was identified in the fermentation/baking module, as significant differences confirmed by Tukey’s test occurred depending on the presence and concentration of the enzyme (1.2; 1.1). In summary, the results of this study indicate that the application of cell-free XynA1 from *C. vibrioides* in bread-making trials can reduce kneading time. It was observed that, after just 10 min of beating, the samples treated with the enzyme showed quantitative improvements in the final product and notably increased in bread height compared to the untreated control (Table 4). These findings are consistent with previously reported results for breads produced with flours supplemented with commercial xylanases [45].

Other studies have also demonstrated improvements in bread characteristics with the addition of xylanases, but all these studies used commercial enzymes [45,46]. Jiang et al. [47] did not use a commercial enzyme but rather a xylanase isolated from *Thermomyces lanuginosus* CAU44 using costly enzymatic purification techniques. In the present work, we used a cell-free extracellular extract (without previous purification) because the *xynA1* gene is more expressed (Figure 5) and the XynA1 enzyme is predominantly secreted in relation to other enzymes (Figure 4) and presents the advantages of low time and cost requirements in complex purification and enzymatic isolation methodologies.

De Queiroz Brito Cunha [48] reported that the enzyme derived from the synthetic gene *xynBS27* of *Streptomyces* sp. encoding the enzyme S27 that was expressed in *Pichia pastoris*, was used in the breadmaking test. The purified S27 was used as an additive in the baking process, promoting a decrease in stiffness and improvements in specific volume. In tests involving the enzymatic treatment of bread with the crude extract of *C. vibrioides* BS-*xynA1*, similar results were obtained for improving bread characteristics.

### 3.8. Alveograph Test

**Figure 8:**
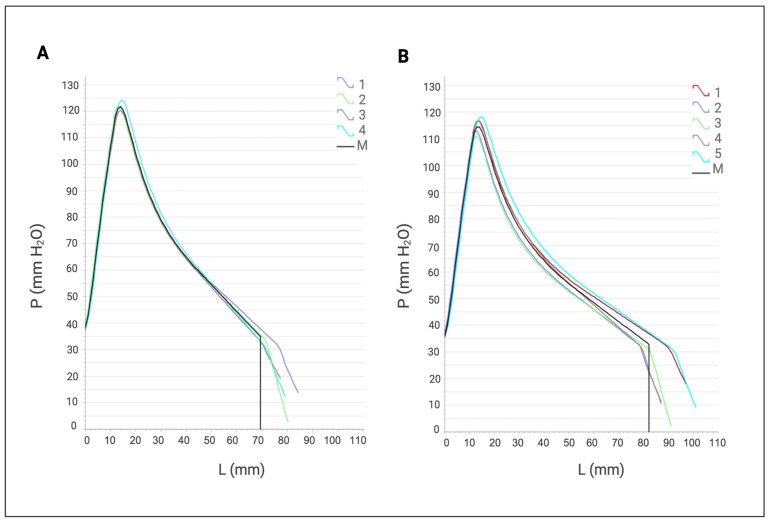
Average alveographs provided by the apparatus (alveograph). (**A**) Control assay. (**B**) Alveographs obtained after treatment with cell-free XynA1 (30 mL of cell-free XynA1 extract in 250 g of flour). The axis of the abscissa shows the values of elasticity (L) expressed in millimeters (mm). The ordinate axis expresses the toughness (P) values expressed in millimeters of water (mmH_2_O). The data represent the arithmetic mean of five independent repetitions. The graph is automatically generated by the AlveoPC/Alveograph software/Chopin Pack.

The alveograph evaluations were carried out through five replicates, both in the absence and presence of the cell-free XynA1. The introduction of the bacterial XynA1 notably improved the dough properties, specifically in terms of elasticity (L) and toughness (P) reduction. This enhancement indicated a favorable impact due to the presence of XynA1, resulting in a dough that is more pliable and less rigid (Figure 8). The addition of XynA1 resulted in increased dough extensibility (L) in tests using the alveograph compared to the control, indicating a significant improvement in dough elasticity while maintaining structural integrity. Furthermore, the addition of XynA1 led to a reduction (from 134 mmH_2_O to 126 mmH_2_O) in toughness (P), effectively reducing dough stiffness (Table 5). In addition, the combined effect of the increased extensibility (L) and decreased toughness (P) resulted in a reduction (from 1.94 to 1.54) in the toughness/elasticity (P/L) ratio compared to the untreated dough. The original graph generated by the alveograph is shown in Figure 8; the numerical data downloaded from the equipment were considered in the statistical analyses shown in Table 5 that show significant differences obtained and confirmed by analysis of variance (Anova) followed by Tukey’s post hoc test. The modular differences calculated for the parameters P, L, and W were lower than the minimum significant limit (LSD) determined by the Tukey test, indicating that the observed variances are statistically different at a level of significance of 99% (*p* < 0.01) (Table 5).

Remaining within the context of the analysis obtained from the alveograph, the addition of XynA1 reduced the maximum pressure (P) and the tensile strength/extensibility ratio (P/L) (Table 5). It is suggested that these results can be attributed to the hydrolysis of insoluble pentosans during the fermentation process, which leads to the release of water and softening of the dough texture. These data are like those observed in *Bacillus subtilis* [49], where the addition of xylanase resulted in better dough consistency during kneading and consequently better bread quality.

**Figure 9:**
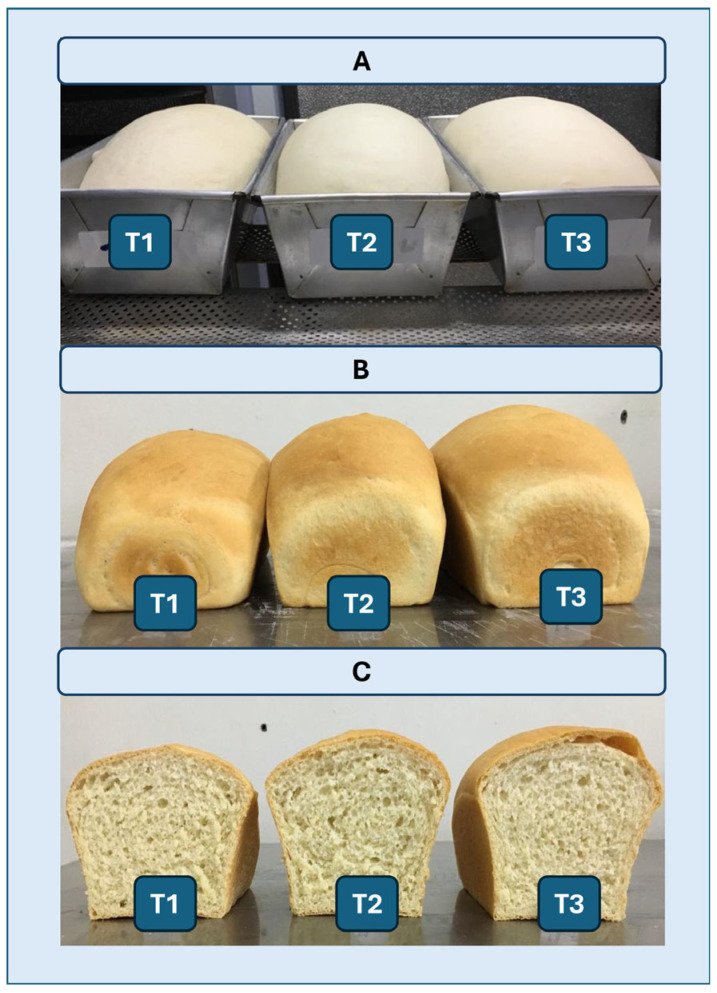
(**A**) Representative images of breads after fermentation. (**B**,**C**) Images of breads after baking. (**B**) Whole breads; (**C**) breads cut exactly in half. (**T1**) Control without the addition of the enzyme extract. (**T2**) Addition of 60 mL of enzyme extract to 1 kg of flour. (**T3**) A total of 120 mL of enzymatic extract containing cell-free XynA1 (specific activity = 278.64 U/mg) was added to 1 kg of flour.

The present study satisfactorily demonstrated that XynA1, when incorporated into the bread-making process, significantly influences the rheological properties of the dough. This influence resulted in an increase in bread volume (Figure 9) and an overall improvement in the alveolar structure of the crumb (Figure 8; Table 4 and Table 5). Furthermore, the present work demonstrated a novel approach to produce XynA1, previously unexplored in this bacterium. The homologous expression in *C. vibrioides* to produce this enzyme followed by biotechnological application represents a groundbreaking advance. Despite of the fact that the results presented contribute to expanding understanding of the potential applications of enzymes produced by the bacterium *C. vibrioides*, opening new avenues for biotechnological innovation and product improvement, it is important to emphasize that the genetic constructs used in this work were developed in a laboratory, which holds NB1/NB2 biosafety certificates. Analyses in this lab using recombinant cells of *C. vibrioides* (NB1) were conducted under containment. Analysis of the enzyme application directly to the bread dough was performed with cell-free XynA1 samples. The enzyme produced is molecularly identical to that produced by the bacteria itself, except that it is more expressed. The strategies developed here are not free for commercial use in Brazil and requires authorization from the National Technical Commission on Biosafety (CTNBio) [50]. In addition, in compliance with Brazilian legislation, no sensory analysis of the breads was performed using applications of products from genetically modified organism (GMO) BS-*xynA1*. The successful application of XynA1 in the baking industry demonstrates its practical and low-cost utility, with potential uses provided they are previously approved by regulatory agencies in Brazil [50].

Recombinant DNA technology and protein engineering offer solutions to common drawbacks associated with native enzymes, such as low yields, inconsistent reproducibility, and suboptimal performance. This allows for the creation of redesigned enzymes with improved properties tailored to specific industrial needs [51]. Although the approach presented in this work is promising, the absence of a purification step can result in the presence of low-molecular-weight secondary metabolites [52], in addition to high-molecular-weight compounds, in the preparation of XynA1 and coexpressed enzymes from *C. vibrioides* (Figure 4). In the absence of a purification process, the composition and concentration of these compounds present in the XynA1-rich cell-free extract can vary considerably, and in practice, the reproducibility of the process may be limited, potentially affecting its industrial applicability. Furthermore, other heat-stable impurities can also affect product quality [53]. To address this drawback, better characterization of potential impurities generated in the secreted extract would be necessary, beyond better characterization of the BS-*xnyA1* secretome using strategies of proteomics associated with LC-MS/MS, which could provide greater safety and reproducibility for future applications.

The alternatives for process improvement for industrial application and obtaining a better product are very broad. It is important to emphasize that cumbersome processes could increase the cost of using the secreted enzyme and undermine the main objective of this work: to propose a low-cost alternative for improving wheat flour, the main raw material for bread. In this sense, it is important to emphasize that bread is one of the most consumed foods in Brazil, a country that still faces high rates of food insecurity [54]. Therefore, proposing lower-cost production alternatives for this food represents not only a technological advancement but also a socially relevant initiative.

In conclusion, the present study demonstrated the successful homologous expression of the *C. vibrioides xynA1* gene and, for the first time, its application as a cell-free enzyme in breadmaking processes. The engineered BS-*xynA1* strain exhibited levels of secreted xylanase activity, with superior specific activity and broad thermal stability when compared to commercially available enzyme mixtures. Functional assays revealed that XynA1 improved dough rheology, increasing extensibility, elasticity, and deformation energy, while reducing toughness and stiffness, resulting in increased bread volume and improved crumb structure. These findings highlight the uniqueness of XynA1 as a biocatalyst with strong potential for industrial baking applications. Prospects include further characterizing the secreted enzyme extract, focusing on optimizing and improving its performance under industrial fermentation conditions, as well as conducting detailed technical and economic analyses to assess its cost-effectiveness relative to commercial enzymes. Further research into the synergistic potential of XynA1 with other hydrolytic enzymes naturally coexpressed in *C. vibrioides* is needed, which could further increase the efficiency of bread dough processing and expand its applicability to other food matrices rich in hemicellulosic substrates. Beyond baking, XynA1’s thermostability and activity profile suggest potential applications in animal feed, bioethanol production, and biomass valorization. Therefore, for commercial use, it is essential to conduct toxicity tests in laboratory models before requesting any authorization from the agencies responsible for approval for use in the food industry. Collectively, the results establish XynA1 as a promising candidate for sustainable biotechnological innovations in the food and bio-based industries.

## Figures and Tables

**Table 1:** Strains and plasmids used in the present report.

Strains/Plasmids	Genotype/Description	Source/Reference
***E. coli***		
DH5*α*	*Δ* *(lacZYA-argF) U169 deoR recA1 endA1 hsdR17 phoA sup144 thi-1 gyrA96 relA1 (* *ϕ* *80 lacZDM15).*	Invitrogen^®^.
S17	M294::RP4-2 (Tc::Mu) (Km::Tn*7*)	[26]
DH5*α*-pAS22-*xynA1*	*E. coli* DH5α carrying pAS22-*xynA1.*	This study
S17-pAS22-*xynA1*	*E. coli* S17 carrying pAS22-*xynA1*	This study
DH5*α*-pAS22	*E. coli* DH5α carrying pAS22	This study
S17-pAS22	*E. coli* S17 carrying pAS22	This study
***C. vibrioides***		
NA1000	Holdfast mutant derivative of wild-type strain CB15	[28]
*BS-xynA1*	NA1000 carrying pAS22-*xynA1*	This study
WT-pAS22	NA1000 carrying pAS22	This study
**Plasmid**		
pAS22	Vector for expression of genes in *Caulobacter* from the P*xylX* promoter, ori T, Cm^R^	[29]
pAS22-*xynA1*	pAS22 containing the *xynA1* gene under the control of P*xylX* promoter	This study

**Table 2:** Test formulations used in the application of xylanase in bread dough preparation.

Components	T1	T2	T3
Flour	1000 g	1000 g	1000 g
Salt	20 g	20 g	20 g
Sugar	60 g	60 g	60 g
Vegetable fat	40 g	40 g	40 g
Yeast	20 g	20 g	20 g
Water	500 mL	440 mL	380 mL
Cell-free XynA1 *	-	60 mL	120 mL

* Cell-free XynA1 activity, 10 U/mL; specific activity, 278.64 U/mg.

**Table 3:** Components used in the preparation of bread dough that was subjected to tests using the alveograph.

Components	XynA1−	XynA1+ *
Flour	250 g	250 g
Water	127.7 mL	97.7 mL
Sodium Chloride	3192.5 g	3192.5 g
Cell-free XynA1 *	no addition	30 mL

* Cell-free XynA1 activity, 10 U/mL; specific activity of XynA1 (278.64 U/mg).

**Table 4:** Parameters checked on breads and statistical analysis of data obtained in the absence and presence of cell-free XynA1 (Anova followed by Tukey’s test).

Parameters		XynA1− (T1)	XynA1+ (T2)	XynA1++ (T3)	
Kneading time to get the veil point (min)		11	10	10	
Height of bread after fermentation (cm)		7.9 ± 0.05	8.6 ± 0.04	9.3 ± 0.05	
Height of bread after baking (cm)		8.8 ± 0.07	9.8 ± 0.08	10.4 ± 0.07	
Anova	Source of Variation	dF	F	*p*-value **	Fc
	Between group	1	980.14	2.05 × 10^−35^	7.15
	Inside group	52			
Tukey Test	Between groups	Ma XynA1− (T1)	Ma XynA1+ (T2)	Ma XynA1++ (T3)	LSD
	Kneading/Fermentation	3.1	1.4	0.7	1.007995
	Fermentation/baking	0.9	1.2	1.1	1.007995
	Kneading/baking	2.2	0.2	0.4	1.007995

**: (*p* < 0.01); dF: degrees of freedom; F: calculated F; Fc: critical F; Ma: modular averages in different groups at different treatment; LSD: least significant difference.

**Table 5:** Results obtained with alveograph before and after treatment with cell-free-XynA1. The mean data are automatically provided by the alveograph platform and are shown in columns 2 and 3. Anova was performed using data obtained from alveograph, and post hoc Tukey test was carried out to compare changes between groups.

Parameters			Anova	F	*p* Value *	Fc	Tukey	LSD
XynA1	−	+	dF = 117	269.38	3.6784 × 10^−79^	2.34	Ma	
P (mmH_2_O)	134	126					8	6.56447705
L (mm)	69	82					13	6.56447705
W (J)	326 × 10^−4^	353 × 10^−4^					27	6.56447705
P/L	1.94	1.54						
eI	53.6%	56.4%						

(−) absence; (+) presence; * *p* < 0.01; dF: degrees of freedom; Ma: difference in the modular means of the tests without and with enzyme (columns 2 and 3); LSD: minimum significant differences defined in Tukey’s test; F: calculated F; Fc: critical F; P: tenacity; L: extensibility; W: mass deformation; P/L: tenacity/extensibility; eI (elasticity index).

## Data Availability

The original contributions presented in this study are included in the article and Supplementary Materials. Further inquiries can be directed to the corresponding author.

## Supplementary Materials

The following supporting information can be downloaded at: https://www.mdpi.com/article/10.3390/microorganisms13102367/s1, Figure S1: Assay to confirm the sterility of enzyme extracts containing XynA1 and used to formulate bread dough.
