# Supplementary material for: Bioengineering Caulobacter vibrioides for Xylanase Applications in the Bakery Industry
Source: Microorganisms. 2025 Oct 15;13(10):2367. doi: 10.3390/microorganisms13102367 (PMC12566495; doi:10.3390/microorganisms13102367)
Supplement: Supplementary file 1 [file microorganisms-13-02367-s001.zip › microorganisms-3856113-supplementary.pptx]

## Slide 1
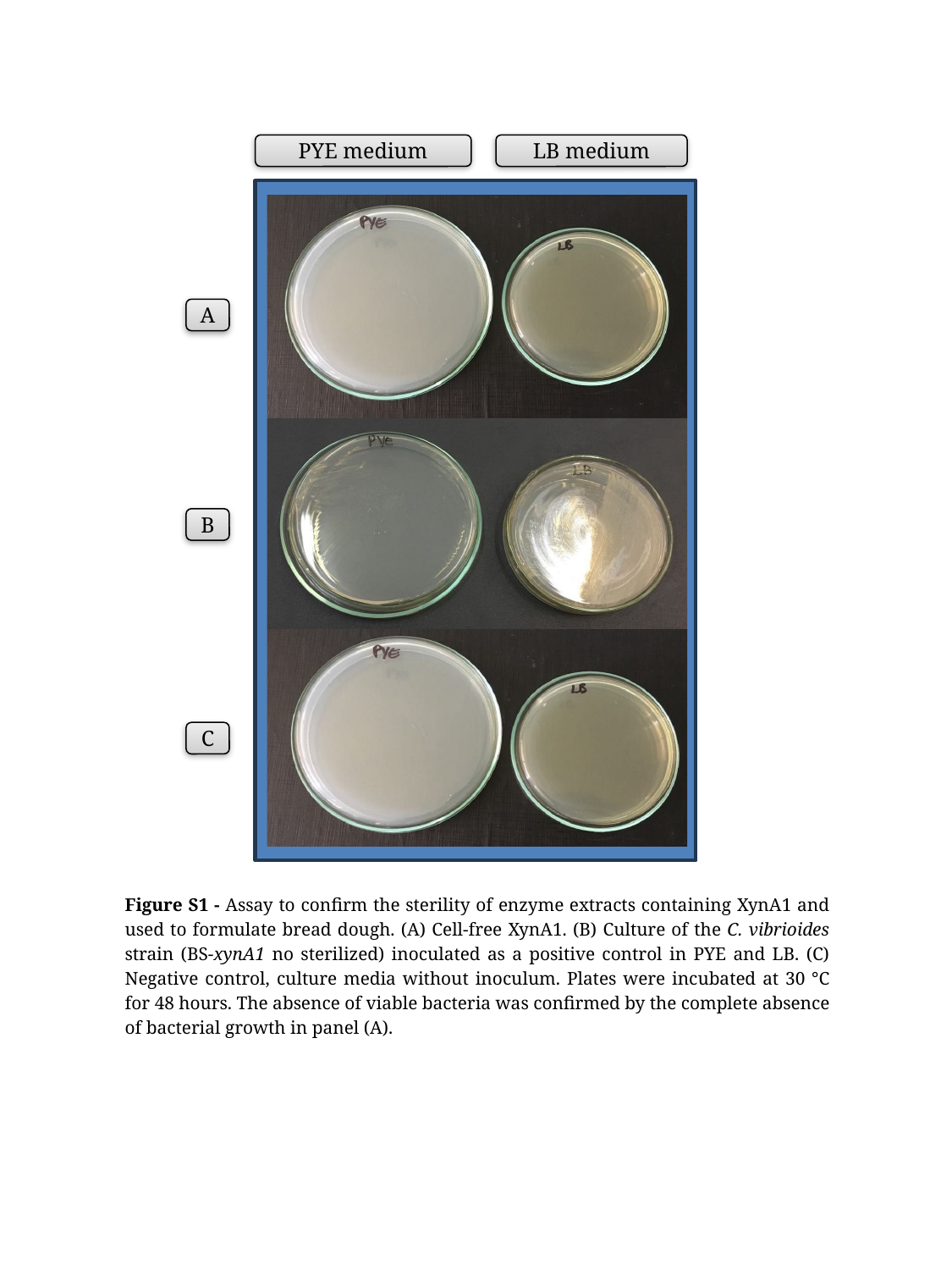

PYE medium
LB medium
A
B
C
Figure S1 - Assay to confirm the sterility of enzyme extracts containing XynA1 and used to formulate bread dough. (A) Cell-free XynA1. (B) Culture of the C. vibrioides strain (BS-xynA1 no sterilized) inoculated as a positive control in PYE and LB. (C) Negative control, culture media without inoculum. Plates were incubated at 30 °C for 48 hours. The absence of viable bacteria was confirmed by the complete absence of bacterial growth in panel (A).
